# Shortcomings of modeling interprofessional collaboration competence in nurse education: A protocol for a scoping review of empirical research

**DOI:** 10.1016/j.mex.2026.104089

**Published:** 2026-08-04

**Authors:** Aldin Striković, Eveline Wittmann, Johannes Krell, Ferdinand Osterhammer

**Affiliations:** a Technical University of Munich, TUM School of Social Sciences and Technology, Department of Educational Sciences, Germany; b Catholic University of Applied Sciences of North Rhine – Westphalia, Germany

**Keywords:** Competence as a continuum, Nurse education, Interprofessional collaboration, Scoping review, Sustainable development goals, Theory talk continuum

## Abstract

Interprofessional collaboration is recognized as central to improving healthcare outcomes. The digital transformation further reshapes collaboration demands, extending the traditional notion of interprofessional collaboration beyond nursing and healthcare. Therefore, interprofessional collaboration competence (ICC) constitutes a key outcome in nurse education. This knowledge-user-informed protocol registered with OSF (*https://osf.io/8rbv7*) outlines a scoping review aiming to identify shortcomings in (1) how competence facets, conceptualized as a continuum (Blömeke et al. 2015), are represented; (2) how nursing students’ ICC is theoretically elaborated, as reflected in the extent of theory talk; and (3) how ICC is empirically modeled in nurse education research. Guided by the JBI methodology and the PRISMA-ScR 2020 guidelines, this scoping review will systematically search the following seven databases to ensure coverage of nursing, educational research, and psychology: (i) Web of Science Core Collection, (ii) Scopus, (iii) ProQuest, (iv) MEDLINE, (v) Education Source, (vi) Cochrane Library, and (vii) APA PsycArticles. By synthesizing the eligible publications, the review seeks to provide a structured overview of research on modeling nursing students’ ICC, thereby enabling the identification of research shortcomings. Completion of the proposed scoping review is anticipated within the period from May to November 2027.

• The proposed review maps which facets of competence-as-a-continuum are addressed in modeling nursing students’ ICC within empirical research.
• It identifies shortcomings in the theoretical and empirical modeling of nursing students’ ICC.
• The findings will provide a basis for future research, informing the development of more effective educational interventions for the ICC of nursing students, and others.

## Graphical abstract

**Figure:**
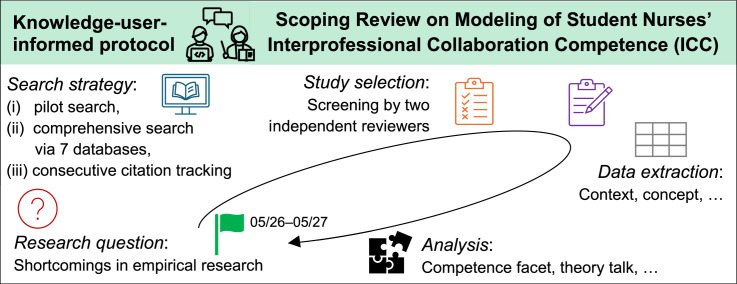

## Specifications table

**Table:** 

**Subject area**	Medicine and Dentistry
**More specific subject area**	*Interprofessional Collaboration Competence in Nurse Education*
**Name of your protocol**	*Shortcomings of modeling interprofessional collaboration competence in nurse education: A protocol for a scoping review of empirical research*
**Reagents/tools**	*Not applicable*
**Experimental design**	*Not applicable. This protocol describes a scoping review.*
**Trial registration**	*This scoping review protocol is registered in the Open Science Framework (OSF)*: https://osf.io/8rbv7
**Ethics**	*For the proposed scoping review, we will use data from publicly available studies. Therefore, ethical approval and informed consent are not required.*
**Value of the Protocol**	• *Addressing a Critical Gap—This protocol proposes a systematic and transparent framework for synthesizing empirical research on modeling nursing students’ interprofessional collaboration competence (ICC)*. • *Identifying Research Shortcomings—The proposed scoping review will provide a theoretically informed mapping of empirical research on modeling nursing students’ ICC in order to identify scientific shortcomings*. • *Guiding Future Research—On the basis of the identified shortcomings, we will propose directions for future research, thereby informing the development of more effective educational interventions to promote the ICC of nursing students, and others*.

## Background

Interprofessional collaboration is widely recognized as a central form of work for improving healthcare outcomes, such as patient safety, and for addressing complex global health challenges as articulated in Sustainable Development Goal 3 ‘Good Health and Well-being’ [[1], [2], [3]]. In addition to collaboration among different healthcare professions, the digital transformation introduces new demands for collaboration between healthcare and technical professions [[4], [5], [6], [7]]. These developments extend the predominant notion of interprofessional collaboration toward broader forms of multi-agency work. In such contexts, collaboration is often not predefined as a specific task but, based on one’s professional role (e.g., autonomy of care recipients), should be initiated in order to develop and implement sustainable solutions in light of societal challenges, such as workforce shortages in healthcare systems [8,9].

Given the significance of interprofessional collaboration, *individual interprofessional collaboration competence* (ICC) constitutes a central learning outcome in nurse education (e.g., [[10], [11], [12]]). However, our previous research—including narrative re-analyses of studies from two meta-analyses [13,14] on simulation-based interprofessional education [9]—suggests that research on modeling nursing students’ ICC may exhibit shortcomings in its theoretical and empirical modeling (see also [11]). To examine this conjecture systematically, a structured synthesis of existing research is required. Although reviews and meta-analyses on specific aspects of ICC exist (e.g., [[15], [16], [17]]; see also [18]), prior searches in major databases (‘Scopus’ and ‘Web of Science’) as well as registries (‘PROSPERO’ and ‘Open Science Framework’) did not show reviews addressing the modeling of nursing students’ ICC—that is, its theoretical conceptualization and operationalization. To fill this gap, we propose a scoping review to map the extent to which facets of competence are addressed in empirical research on nursing students’ ICC, thereby enabling the identification of research shortcomings in the field (see [[19], [20], [21]]). This will help to identify avenues for future research that can inform the development of more effective educational interventions for facilitating the ICC of nursing students, and others.

### Description of protocol

This scoping review protocol is based on the Preferred Reporting Items for Systematic Reviews and Meta-Analyses Protocols (PRISMA-P: [22,23]; see Appendix A) and the JBI (previously Joanna Briggs Institute) methodology for conducting systematic scoping reviews ([[24], [25], [26]]; for examples, see [[27], [28], [29]]). In this protocol, we outline the (1) research question and the (2) inclusion criteria. Furthermore, we describe the (3) proposed approach to evidence searching, selection, data extraction, and analysis. The ensuing scoping review will adhere to the Preferred Reporting Items for Systematic Reviews and Meta-Analyses extension for Scoping Reviews (PRISMA-ScR) checklist [30].

### Research question and theoretical framework

In the proposed scoping review, the following research question will inform the development of inclusion criteria and guide the systematic analysis of included literature:

*What shortcomings are evident in the modeling of nursing students’ interprofessional collaboration competence (ICC) within empirical research?*

**Fig. 1:**
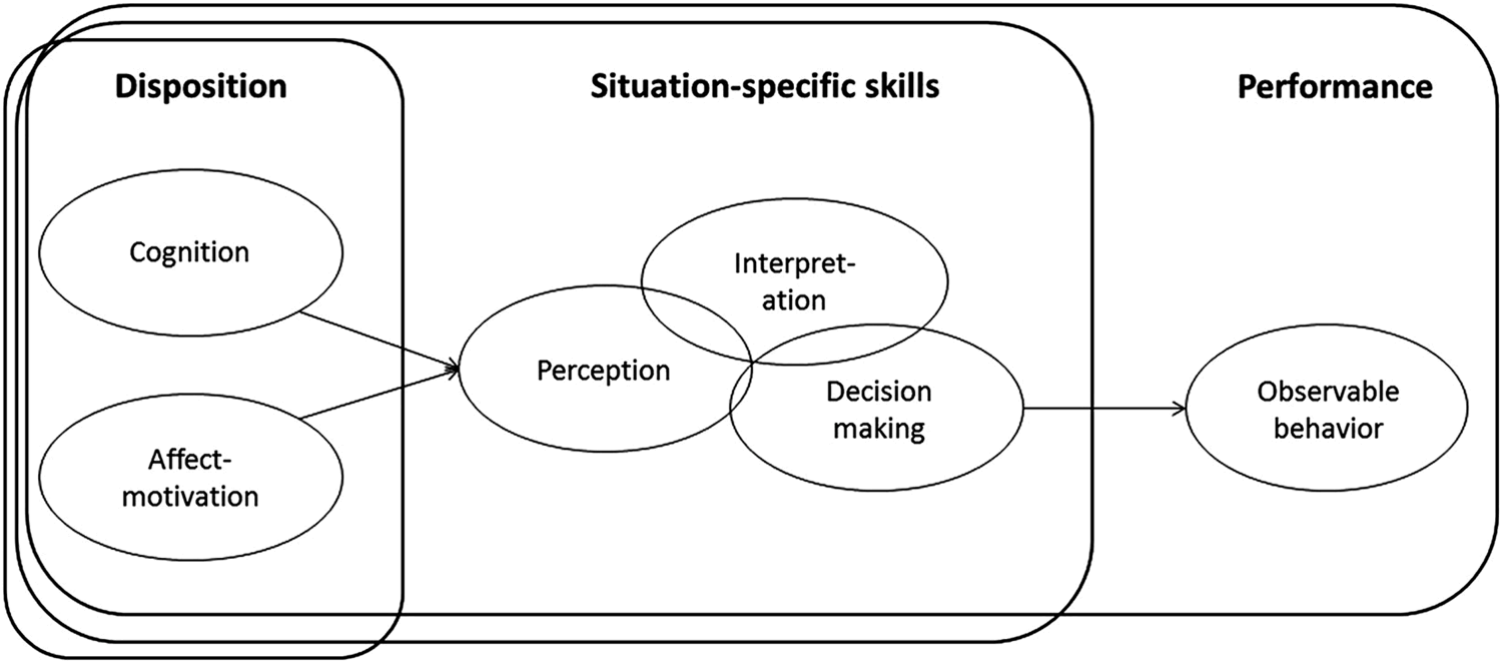
*Competence as a continuum*. Used with permission from *Zeitschrift für Psychologie* 2015; 223 (1): 3-13; https://doi.org/10.1027/2151-2604/a000194 © 2015 Hogrefe Publishing www.hogrefe.com.

To theoretically ground our research question, we situate it within a comprehensive conceptualization of competence that delineates a continuum from relatively stable “traits (cognitive, affective, motivational) that underlie the perception, interpretation, and decision-making that give rise to observed behavior in a particular real-world situation” ([31], p. 11). Drawing on contemporary psychological theories and measurement approaches, Blömeke et al. [31] proposed the competence-as-a-continuum model (see Fig. 1) to account for both the stability and variability of individuals across situations—that is, the person–situation relation.

The model hypothesizes a causal linkage between different latent facets of competence and observable behavior along a continuum defined by their predominant levels of situatedness. Within this framework, relatively stable patterns of thought, affect and motivation form the basis for situation-specific cognitive processes, which in turn determine the likelihood of particular behaviors in specific situations (see also [32]). In the context of the proposed scoping review, this model serves as a theoretical lens to systematically map the facets of competence addressed in research on nursing students’ ICC, thereby identifying aspects in need of further development.

### Selection criteria

*Population, concept and context*: In accordance with the JBI methodology for scoping reviews, we will use the framework of population, concept, and context (PCC) to identify the relevant studies [25,26]. The PCC framework provides structured guidance for formulating research questions and defining selection criteria for a scoping review (see [33]). In the proposed scoping review, the population of interest is represented by nursing students, with their ICC as the focal concept. The context encompasses various settings (e.g., higher education, vocational education and training, continuing education, etc.), without geographical or publication-date restrictions.

*Types of evidence sources*: The scoping review will include empirical studies regardless of methodological approach, study design, or publication type, whether peer-reviewed or not. Non-English, Non-German or unavailable full-text publications will be excluded.

The criteria for selecting the articles are listed as inclusion requirements (Table 1).

**Table 1:** Inclusion criteria.

Selection schema	Specification as inclusion requirements
Population	Nursing students
Concept	All facets of competence as a continuum (see Fig. 1)
Context	All types of settings (e.g., higher education, vocational education and training)
Types of evidence sources	Empirical studies (i.e., qualitative, quantitative, and mixed methods)
	All types of study design (e.g., observational, experimental, longitudinal)
	All publication types (e.g., journal articles, conference proceedings)
	English-language and German-language records
	Full text available

### Search strategy

Following the JBI methodology for scoping reviews, the search strategy is based on a three-step approach: (1) a pilot search, (2) the comprehensive search, and (3) citation tracking [25,34].

*(1) Pilot search*: This step comprised successive, limited searches conducted in at least the Cochrane Library, Web of Science Core Collection, Scopus, and MEDLINE. Analyses of the records retrieved in each search served to iteratively refine the search query. Its interim versions and the interdisciplinary selection of databases were discussed on several occasions in academic discourse, for example at a scientific conference [35] and in research colloquia with research colleagues in the field of educational assessment and/or nurse education research, some of whom also have prior experience in nurse education, professional nursing practice, or both. As “those invested in the production of research, and who may benefit or be impacted by research” ([36], pp. 969–970), these colleagues contributed ‘knowledge user’ perspectives to the search strategy. With respect to the pilot versions of the search string, it was suggested to include related terms such as ‘interdisciplinary’ to maximize relevant records, since terminology in the literature on interprofessional collaboration is not used consistently (see [37]). Furthermore, it was recommended to restrict the search to titles, abstracts, and keywords in order to increase specificity—that is, the ratio of relevant records to the total publication count (see [38]). Using the entire publication text as a search field would have mainly yielded additional publications in which the search terms are scattered throughout the text but do not appear in a manner relevant to answering the research question (see [39]). In addition, colleagues with content expertise occasionally referred to relevant studies that were not retrieved by earlier versions of the search query; these studies were used to evaluate and further refine the search strategy (see [34]).

*(2) Comprehensive search*: Based on the iterative pilot search, the following basic string was derived and is used to ensure a comprehensive search strategy across seven databases (see Table 2):

("interprofessional cooperation" OR "interprofessional cooperative" OR "interprofessional collaboration" OR "interprofessional collaborative" OR "inter-professional cooperation" OR "inter-professional cooperative" OR "inter-professional collaboration" OR "inter-professional collaborative" OR "multi-professional collaboration" OR "multi-professional collaborative" OR "multiprofessional collaboration" OR "multiprofessional collaborative" OR "multiprofessional cooperation" OR "multiprofessional cooperative" OR "multi-professional cooperation" OR "multi-professional cooperative" OR "interdisciplinary cooperation" OR "interdisciplinary cooperative" OR "interdisciplinary collaboration" OR "interdisciplinary collaborative" OR "inter-disciplinary cooperation" OR "inter-disciplinary cooperative" OR "inter-disciplinary collaboration" OR "inter-disciplinary collaborative" OR "multi-disciplinary collaboration" OR "multi-disciplinary collaborative" OR "multidisciplinary collaboration" OR "multidisciplinary collaborative" OR "multidisciplinary cooperation" OR "multidisciplinary cooperative" OR "multi-disciplinary cooperation" OR "multi-disciplinary cooperative" OR "crossprofessional cooperation" OR "crossprofessional cooperative" OR "crossprofessional collaboration" OR "crossprofessional collaborative" OR "cross-professional cooperation" OR "cross-professional cooperative" OR "cross-professional collaboration" OR "cross-professional collaborative" OR "transprofessional cooperation" OR "transprofessional cooperative" OR "transprofessional collaboration" OR "transprofessional collaborative" OR "trans-professional cooperation" OR "trans-professional cooperative" OR "trans-professional collaboration" OR "trans-professional collaborative" OR "crossdisciplinary collaboration" OR "crossdisciplinary collaborative" OR "crossdisciplinary cooperation" OR "crossdisciplinary cooperative" OR "cross-disciplinary collaboration" OR "cross-disciplinary collaborative" OR "cross-disciplinary cooperation" OR "cross-disciplinary cooperative" OR "transdisciplinary collaboration" OR "transdisciplinary collaborative" OR "transdisciplinary cooperation" OR "transdisciplinary cooperative" OR "trans-disciplinary collaboration" OR "trans-disciplinary collaborative" OR "trans-disciplinary cooperation" OR "trans-disciplinary cooperative") AND (competenc* OR capacit* OR capabilit* OR abilit* OR skill*) AND nurs*

**Table 2:** Search strategy and filtered publication counts by database.

Database (interface) [Coverage]	Query	Records May 25, 2026 [Filters]
Scopus (dto.) [1974–2026]	TITLE-ABS-KEY[Basic string]	1.907 [English; German]
MEDLINE (Clarivate) [1985–2026]	TS=[Basic string]	1.654 [English; German]
Web of Science Core Collection (Clarivate) [1988–2026]	TS=[Basic string]	1.570 [English; German]
ProQuest (dto.) [1989–2026]	TIAB[Basic string]	355 [English; German]
Education Source (EBSCO) [1989–2026]	AB [Basic string] OR KW [Basic string] OR TI [Basic string]	194 [English; German]
Cochrane Library (Ovid) [2000–2026]	[Basic string].ab. or [Basic string].ti. or [Basic string].kw.	77 [English; German]
APA PsycArticles (EBSCO) [1989–2024]	AB [Basic string] OR KW [Basic string] OR TI [Basic string]	3 [English]
**Total**	Not deduplicated	5.760

Note: Adaptations with controlled vocabulary, such as MeSH terms, were examined (see Appendix B). As they did not generate any additional relevant records beyond the basic string, the comprehensive search is based on the more parsimonious basic string to enhance reproducibility.

*(3) Consecutive searching via citation tracking*: According to the JBI methodology for scoping reviews, the third step of searching typically involves examining reference lists [25,34]. In the proposed scoping review, this backward citation tracking will be applied to all relevant studies identified by the primary search (see Table 2). In addition, we will search for citing records (i.e., forward citation tracking; see [40]). Both procedures will be semi-automatically and simultaneously conducted using *citationchaser* (see [41]; https://estech.shinyapps.io/citationchaser/)—a tool based on the lens.org database aggregator, which includes >300 million scholarly records (May 2026). Citation tracking will be repeated iteratively until no additional eligible studies are identified.

*Conditional gray literature searching*: A targeted search for gray literature—sources that are often not indexed in traditional academic databases—is not necessarily considered essential for grasping the *empirically* focused facets of the subject matter of interest (i.e., nursing students’ ICC) and the associated research shortcomings. However, if the proposed search strategy, contrary to expectations arising from the pilot searches, does not yield a ‘large scoping review’ (defined as > 100 eligible studies; [42]), we will conduct a targeted gray literature search using Google Scholar and ClinicalTrials.gov. In addition, the knowledge users mentioned in the ‘*(1) Pilot search*’ paragraph will be consulted regarding complementary sources, such as conference proceedings. All gray literature resources searched, access dates, and adapted search strategies will be documented and appended to the final scoping review (see [43]).

### Study selection

**Fig. 2:**
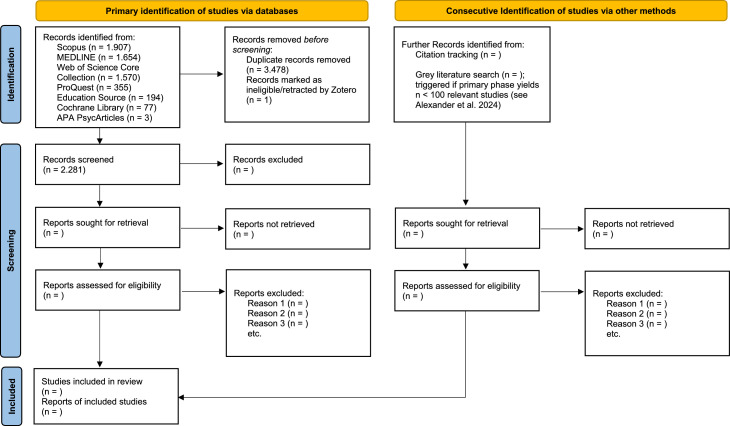
PRISMA flow diagram.

All records identified through the proposed search strategy will be imported into the reference management software Zotero for deduplication. The selection process will proceed in two stages. First, using ASReview [44], two independent reviewers will screen titles and abstracts to assess eligibility based on the inclusion criteria (see Table 1). Second, available full texts of potentially relevant studies will be retrieved and evaluated by the same reviewers against the inclusion criteria. Prior to both screening phases, the reviewers will conduct a pilot screening using a random sample of 30 and 15 records, respectively, to ensure consistency and clarity of the procedure. To support both screening phases, the authors developed Python scripts (see Appendix C) and tested them using files from pilot studies or dummy data. Based on CSV files exported from ASReview and/or Zotero, these scripts semi-automatically calculate reviewer agreement using Cohen’s kappa and identify records for which reviewers’ decisions diverge. Any disagreements during the selection process will be resolved through discussion; if consensus cannot be reached, a third researcher will act as an adjudicator. The outcomes of the search and selection process will be documented and visualized in a PRISMA flow diagram (Fig. 2), which details the stages of identification, screening, and inclusion, culminating in the final number of included studies [45].

### Data extraction

One reviewer will extract the data from the included studies, with a second reviewer verifying the accuracy of the extracted data (see [42]). For this purpose, we will use a structured data extraction form that includes established extraction categories (e.g., authors, year of publication), the PCC framework, and the competence-as-a-continuum model (see Fig. 1). Table 3 presents a draft version of the extraction framework; the draft of the data extraction form is provided in Appendix D, together with completed examples derived from pilot studies. Should additional detail be required to address the research question, the extraction process may be refined. All changes will be systematically documented and reported within the scoping review.

**Table 3:** Data extraction framework.

Extraction category	Specifications
*Author(s) (year of publication)*	e.g. Marion-Martins and Pinho [13]
*Study design (methodological approach)*	e.g., experimental (quantitative)
*Study aim(s)*	e.g. to evaluate an intervention
*Population*	Group and size (e.g., 25 nursing students and 15 dental hygiene students)
*Context*	Country (i.e., location of the study)
	Setting (e.g., higher education)
*Concept*	Focused facet(s) of competence (e.g., affect-motivation; see Fig. 1)
	Instrument if applicable (e.g., the Interprofessional Collaborative Competencies Attainment Survey of [46,47]; the Interprofessional Collaborator Assessment Rubric of [48]; the IPEC Competency Self-Assessment Survey of [49,50])
	Extent of theory talk (None = at most framework-related; Low = theory dropping or theory relating; High = theory application or theory generation; see [51,52])
	Qualitative findings pertinent to the modeling of ICC

Note: Non-applicable extraction items will be coded as ‘Not applicable’; missing information will be recorded as ‘Not reported’ (see also Appendices D and E).

Given the expected volume of >100 eligible studies (see *Search strategy* section), the first reviewer will extract data using Gemini Notebook (formerly NotebookLM) while maintaining extraction quality through a human-in-the-loop workflow (i.e., human verification). Drawing on recent research on AI-assisted data extraction and corresponding prompt engineering strategies (e.g., [53,54]), we developed a Gemini Notebook prompt that operationalizes the same completion instructions applied by the human reviewers (see Appendix E)—except for the extent of theory talk.

### Quality assessment

In accordance with the methodological framework for scoping reviews (e.g., [33]), a comprehensive critical appraisal of the included studies will not be conducted, as study quality does not affect the primary aim of mapping empirical research on modeling nursing students’ ICC. Nevertheless, to further gauge possible causes of shortcomings within empirical research (see [55]), we will assess the extent to which theory is employed by researchers (see the last specification in Table 3). For this purpose, we will apply an adapted version of the theory-talk coding scheme informed by Lyons et al. [52]; for further examples of such measures, see [51,56,57]]:

• ‘None’ is assigned when the study mentions no theory and/or only refers to one or more frameworks (e.g., [58,59]). Frameworks are understood as loosely connected sets of premises and constructs that provide a distinct lens through which the phenomenon of interest can be examined. However, they lack propositions required for explanations and/or predictions of empirically observable phenomena (i.e., theory, see [60,61]).
• The code ‘Low’ is applied when the study demonstrates theory dropping or theory relating. Theory dropping occurs when a theory is mentioned in the introduction or methods section without revisiting it later in the study. Theory relating occurs when a theory is used in the discussion to interpret and/or contextualize findings but does not meaningfully inform the study design or data analysis.
• A study receives a ‘High’ rating if it demonstrates theory application or theory generation. Theory application involves integrating a theory throughout the study to guide both design and analysis. This may include empirically corroborating a theory’s propositions, while theory generation focuses on building, refining, or extending a theory.

The coding framework will be pilot-tested by two reviewers to ensure consistency and reliability in evaluating the extent of theory talk in each included record. Any disagreements between reviewers will be resolved through discussion or, if necessary, with the involvement of a third reviewer.

### Data analysis and presentation

To identify shortcomings in modeling nursing students’ ICC within empirical research, the *data analysis* will be twofold (see [25). First, deductively extracted data will be analyzed using descriptive statistics, such as the frequencies and percentages of the competence-facet assignments across the included studies (i.e., cognition, affect-motivation, situation-specific skills, and performance; [31,62]). Second, the extraction items regarding study aim(s) and qualitative findings pertinent to the modeling of ICC (see Table 3) will be “categorized into high level themes to enable the reader to make sense of the results using simple content analysis” ([63], p. 180; see also [33]). For this purpose, inductive categories will be developed on the basis of the extracted qualitative data. Once these categories have been consolidated by the review team, two reviewers will independently assign the extracted data to the categories. Any disagreements will be resolved through discussion and, if necessary, consultation with a third reviewer.

**Fig. 3:**
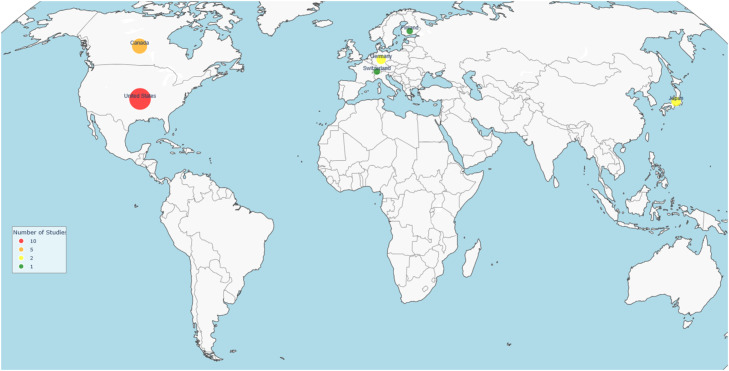
Example of a world heat map showing the number of included studies by country (generated with Plotly).

**Fig. 4:**
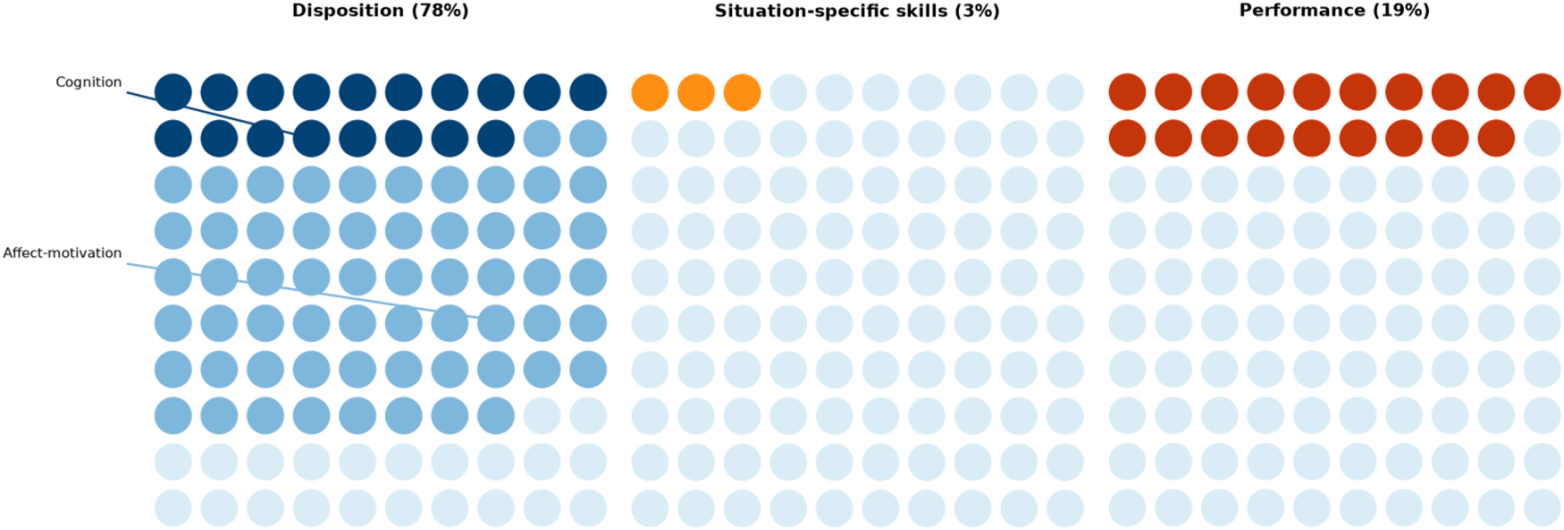
Example of a waffle chart showing the coverage of the competence-as-a-continuum model across the included studies (generated with Plotly). Due to their highly interrelated, process-oriented character, the PID components will be subsumed by ‘Situation-specific skills’ as a situated cognitive facet of competence ([62]; see also Appendix E).

With regard to the *presentation of results*, current plans include, for instance, visualizing the geographical distribution of studies through a world heat map (e.g., Fig. 3). Furthermore, coverage of the competence-as-a-continuum model (see Fig. 1) across the included studies will be visualized using a waffle chart differentiated by competence facets (see Fig. 4). To further elaborate the research question concerning shortcomings in the modeling of nursing students’ ICC, a cross-tabulation is proposed relating the frequency of competence facets to theory-talk codings, with the instruments presented as subgroups within each competence facet. All visualizations will be accompanied by a narrative to relate the findings to the research question. While the heterogeneity of the included studies will be acknowledged, our theoretically informed approach, particularly by conceptualizing competence as a continuum (e.g., [31]), provides a common analytical framework for comparing diverse studies (for the broad nature of scoping reviews, see [21,25]). In line with Khalil and Tricco [63], the narrative summary will be structured around the findings of the descriptive statistics and the qualitative content analysis to help readers make sense of the results.

Reporting will adhere to the PRISMA-ScR guideline [30], to ensure transparency and reproducibility. After the scoping review has been accepted for publication, all metadata files from the databases will be made publicly available as supplementary material in RIS format via the OSF repository (https://osf.io/). Should the scoping review be updated, any newly generated supplementary datasets will be deposited in the same repository.

### Timeline

Table 4 outlines our 12-month timeline, structured according to the nine steps of JBI-guided scoping reviews [21,64], and specifies the review team members responsible for each review stage.

**Table 4:** Proposed timeline for conducting the scoping review (for an example, see [39]).

Stage	Task [specialized	Timeframe	Responsible team member(s)
1	Defining and aligning the objective(s) and question(s)	—	AS, EW
2	Developing and aligning the inclusion criteria with the objective(s) and question(s)	—	AS, EW
3	Describing the planned approach to evidence searching, selection, extraction, analysis, and presentation	—	AS, EW, JK, FO
4	Searching for the evidence (Zotero software for storage of search exports and deduplication)	Months 0–1	AS
5	Study selection (ASReview for title and abstract screening by two independent reviewers; Zotero software for documenting full-text screening decisions and exclusion reasons)	Months 0–3	AS, FO
6	Data extraction (see Table 3) assisted by Gemini Notebook	Months 3–5	AS, JK
7	Data analysis	Months 5–8	AS, EW, JK, FO
8	Presentation of the results	Months 8–11	AS, EW, JK, FO
9	Summarizing the evidence in relation to the purpose of the review, making conclusions, and noting any implications of the findings	Months 11–12	AS, EW, JK, FO

Note: Stages 1–3 precede the scoping review (see [64]). Therefore, no timeframe is specified for these stages.

*Team composition:* AS and EW draw on previous research on the theoretical and empirical modeling of competencies in nurse education, particularly nursing students’ ICC (e.g., [65,66]). To mitigate the risk that preconceptions arising from this line of research (e.g., [9]) distort the scoping review and its findings, the review team also includes JK, Professor of nurse education, and FO, lecturer in nurse education—neither of whom has been involved in previous research conducted by AS and EW on the modeling of nursing students’ ICC. To ensure such an independent perspective throughout the review process, JK and/or FO will be involved across all relevant subsequent stages of the scoping review (i.e., study selection, data extraction, analysis, synthesis, and interpretation of findings). Furthermore, while AS possesses methodological knowledge and experience in conducting the stage-specific tasks of scoping reviews (e.g., [35]), additional support from an academic librarian may be sought when necessary to complement the review team’s expertise in information retrieval and search procedures.

*Contingency plans for delays:* Should any contingencies occur, stage-specific responsible team members may, where feasible, be supported by additional members of the review team and/or by colleagues from the respective research group at the Technical University of Munich (TUM). However, such contingency measures for adhering to the proposed timeline will not be taken if they are likely to compromise the methodological rigor. Instead, the timeline will be adjusted where necessary to maintain the quality of the scoping review. If, before manuscript submission, >12 months have elapsed since the last comprehensive search, monthly update checks will be conducted using Scopus and Web of Science (i.e., Core Collection and MEDLINE). In line with the arguments of Stokes et al. [67], the decision to conduct a full search update will be guided by whether newly published studies are likely to meaningfully affect the review findings rather than by the “one-size-fits-all ’12-month rule’” (p. 359). Specifically, we will examine whether newly identified studies merely provide additional examples of patterns already identified in the scoping review or indicate substantive changes in how nursing students’ ICC is modeled. If the latter is the case, we will fully update the search in accordance with this scoping review protocol. Consequently, the overall timeline for conducting the scoping review would be expected to extend from 12 to approximately 18 months (i.e., May–Nov. 2027).

## Protocol validation

### Knowledge user consultation

With regard to JBI guidance on knowledge user engagement in scoping reviews ([36]; see also [68]), several consultation activities are noteworthy. Through these activities, we sought to obtain information and feedback from knowledge users on scoping review stages 1–3 of the proposed scoping review (see Table 4). As already indicated in the *Search strategy* section, the consulted knowledge users constituted a convenience sample of research colleagues in the fields of educational assessment and/or nurse education research, including research on interprofessional collaboration, who initially emphasized the relevance of the proposed scoping review (see stage 1 in Table 4). Subsequent consultation activities comprised three semi-formal research colloquia (approximately 1–2 h each), with discussions centered on presentations of draft versions of review stages (for further examples of consultation methods, see [68,69]).

Corresponding to stage 2 of the JBI scoping review process (see Table 4), the first research colloquium focused on inclusion criteria. To ensure that the scoping review is meaningful to the international community, the knowledge users recommended extending the scope beyond non-academic nurse education to also include academic nurse education (see Table 1). The second research colloquium focused on the approach to evidence searching (see stage 3 in Table 4). Knowledge users provided feedback on a draft version of the search query, including recommendations on terminology, database selection, and search-field restrictions; further recommendations were given by researchers at an educational research conference, including individuals experienced in evidence synthesis. As described in the *Search strategy* section, this feedback contributed to several refinements of the search string. Following these refinements, a pilot version of the scoping review was conducted to obtain feedback on a preliminary data extraction form. While the consulted knowledge users acknowledged the value of an initially broad extraction approach, they recommended aligning the data extraction more closely with the review question (see Appendix D).

### Protocol registration and amendments

Based on literature searches, we could not identify any reviews directly addressing the research question of this proposed scoping review, thereby underscoring its ongoing relevance and necessity. The review will be conducted according to the JBI methodology for scoping reviews (e.g., [33]) and reported in accordance with the PRISMA-ScR guidelines [30]. Their application ensures a systematic, transparent, and reproducible approach throughout the review process. The protocol is registered via the ‘Generalized Systematic Review’ form [70] on Open Science Framework (https://osf.io/8rbv7). Any methodological refinements considered necessary during the conduct of the review will be documented transparently as protocol amendments in the corresponding OSF registration.

## Limitations

Despite careful efforts to minimize limitations of this protocol, certain limitations remain. Restricting the search to English- and German-language publications may result in the exclusion of relevant studies published in other languages, which could lead to geographic bias. Furthermore, given the already comprehensive search strategy, gray literature will not be included in the initial search. Nevertheless, this is acknowledged as a potential limitation. To mitigate this, gray literature will be considered if, contrary to expectations arising from the pilot searches, the evidence searching does not yield a large scoping review (i.e., > 100 eligible studies; [42]). Given the anticipated workload associated with a large scoping review and the resources available to the review team, knowledge user engagement beyond the protocol development is not considered feasible (see [36]). Finally, as is typical for scoping reviews, the proposed scoping review will synthesize the available evidence without formally assessing the methodological quality of included studies, thereby limiting the ability to draw quantitative conclusions about the strength of the evidence base. These limitations highlight potential avenues for future research that may further advance understanding of nursing students’ ICC.

## Declaration of generative AI and AI-assisted technologies in Python code development

During the preparation of the Python scripts provided in Appendix C and the example visualizations presented in Fig. 3, Fig. 4, the authors used ChatGPT to support code development. The scripts in Appendix C are intended to assist with the title and abstract screening and the full-text screening stages. Based on CSV files exported from ASReview and/or Zotero, these scripts semi-automatically calculate intercoder agreement using Cohen’s kappa and identify records for which reviewers’ decisions diverge. The authors tested the scripts with files from pilot studies and/or dummy data. Following testing and reviewing, the authors take full responsibility for the functionality of the appended Python scripts and for the accuracy of the Plotly-based visualizations to support the proposed scoping review.

## CRediT authorship contribution statement

**Aldin Striković:** Conceptualization, Data curation, Methodology, Project administration, Software, Visualization, Writing – original draft, Writing – review & editing. **Eveline Wittmann:** Conceptualization, Methodology, Supervision, Writing – review & editing. **Johannes Krell:** Conceptualization, Methodology, Project administration, Writing – review & editing. **Ferdinand Osterhammer:** Conceptualization, Methodology, Project administration, Writing – review & editing.

## Declaration of competing interest

The authors declare that they have no known competing financial interests or personal relationships that could have appeared to influence the work reported in this paper.

## Data Availability

Data will be made available on request.

## Appendix Supplementary materials

Appendix A. A_PRISMA_P Checklist Image, application 1

Appendix B. B_Comparison of database-specific search strategies with and without controlled vocabulary Image, application 2

Appendix C. C_Python scripts supporting intercoder agreement – Python 3.14.5 Image, application 3

Appendix D. D_Data extraction form with completed examples from pilot studies Image, application 4

Appendix E. E_Data extraction prompt Image, application 5
