# Supplementary material for: Shortcomings of modeling interprofessional collaboration competence in nurse education: A protocol for a scoping review of empirical research
Source: MethodsX. 2026 Aug 4;17:104089. doi: 10.1016/j.mex.2026.104089 (PMC13474387; doi:10.1016/j.mex.2026.104089)
Supplement: Supplementary file 2 [file mmc2.docx]

**Comparison of database-specific search strategies with and without controlled vocabulary**

| **Database** | **S1: Database-specific adapted search string with controlled vocabulary** | **S2: Database-specific query using the basic string (see Table 2)** | **Records* for**  **S1 NOT S2**  ***Relevant* Records for S1 NOT S2** |
| --- | --- | --- | --- |
| MEDLINE | (((MT=(Interdisciplinary Communication)) OR (MT=(Physician-Nurse Relations)) OR (TS=("interprofessional cooperation" OR "interprofessional cooperative" OR "interprofessional collaboration" OR "interprofessional collaborative" OR "inter-professional cooperation" OR "inter-professional cooperative" OR "inter-professional collaboration" OR "inter-professional collaborative" OR "multi-professional collaboration" OR "multi-professional collaborative" OR "multiprofessional collaboration" OR "multiprofessional collaborative" OR "multiprofessional cooperation" OR "multiprofessional cooperative" OR "multi-professional cooperation" OR "multi-professional cooperative" OR "interdisciplinary cooperation" OR "interdisciplinary cooperative" OR "interdisciplinary collaboration" OR "interdisciplinary collaborative" OR "inter-disciplinary cooperation" OR "inter-disciplinary cooperative" OR "inter-disciplinary collaboration" OR "inter-disciplinary collaborative" OR "multi-disciplinary collaboration" OR "multi-disciplinary collaborative" OR "multidisciplinary collaboration" OR "multidisciplinary collaborative" OR "multidisciplinary cooperation" OR "multidisciplinary cooperative" OR "multi-disciplinary cooperation" OR "multi-disciplinary cooperative" OR "crossprofessional cooperation" OR "crossprofessional cooperative" OR "crossprofessional collaboration" OR "crossprofessional collaborative" OR "cross-professional cooperation" OR "cross-professional cooperative" OR "cross-professional collaboration" OR "cross-professional collaborative" OR "transprofessional cooperation" OR "transprofessional cooperative" OR "transprofessional collaboration" OR "transprofessional collaborative" OR "trans-professional cooperation" OR "trans-professional cooperative" OR "trans-professional collaboration" OR "trans-professional collaborative" OR "crossdisciplinary collaboration" OR "crossdisciplinary collaborative" OR "crossdisciplinary cooperation" OR "crossdisciplinary cooperative" OR "cross-disciplinary collaboration" OR "cross-disciplinary collaborative" OR "cross-disciplinary cooperation" OR "cross-disciplinary cooperative" OR "transdisciplinary collaboration" OR "transdisciplinary collaborative" OR "transdisciplinary cooperation" OR "transdisciplinary cooperative" OR "trans-disciplinary collaboration" OR "trans-disciplinary collaborative" OR "trans-disciplinary cooperation" OR "trans-disciplinary cooperative"))) AND (TS=(competenc* OR capacit* OR capabilit* OR abilit* OR skill*)) AND (MT=Students, Nursing)) | (TS=(("interprofessional cooperation" OR "interprofessional cooperative" OR "interprofessional collaboration" OR "interprofessional collaborative" OR "inter-professional cooperation" OR "inter-professional cooperative" OR "inter-professional collaboration" OR "inter-professional collaborative" OR "multi-professional collaboration" OR "multi-professional collaborative" OR "multiprofessional collaboration" OR "multiprofessional collaborative" OR "multiprofessional cooperation" OR "multiprofessional cooperative" OR "multi-professional cooperation" OR "multi-professional cooperative" OR "interdisciplinary cooperation" OR "interdisciplinary cooperative" OR "interdisciplinary collaboration" OR "interdisciplinary collaborative" OR "inter-disciplinary cooperation" OR "inter-disciplinary cooperative" OR "inter-disciplinary collaboration" OR "inter-disciplinary collaborative" OR "multi-disciplinary collaboration" OR "multi-disciplinary collaborative" OR "multidisciplinary collaboration" OR "multidisciplinary collaborative" OR "multidisciplinary cooperation" OR "multidisciplinary cooperative" OR "multi-disciplinary cooperation" OR "multi-disciplinary cooperative" OR "crossprofessional cooperation" OR "crossprofessional cooperative" OR "crossprofessional collaboration" OR "crossprofessional collaborative" OR "cross-professional cooperation" OR "cross-professional cooperative" OR "cross-professional collaboration" OR "cross-professional collaborative" OR "transprofessional cooperation" OR "transprofessional cooperative" OR "transprofessional collaboration" OR "transprofessional collaborative" OR "trans-professional cooperation" OR "trans-professional cooperative" OR "trans-professional collaboration" OR "trans-professional collaborative" OR "crossdisciplinary collaboration" OR "crossdisciplinary collaborative" OR "crossdisciplinary cooperation" OR "crossdisciplinary cooperative" OR "cross-disciplinary collaboration" OR "cross-disciplinary collaborative" OR "cross-disciplinary cooperation" OR "cross-disciplinary cooperative" OR "transdisciplinary collaboration" OR "transdisciplinary collaborative" OR "transdisciplinary cooperation" OR "transdisciplinary cooperative" OR "trans-disciplinary collaboration" OR "trans-disciplinary collaborative" OR "trans-disciplinary cooperation" OR "trans-disciplinary cooperative") AND (competenc* OR capacit* OR capabilit* OR abilit* OR skill*) AND nurs*)) | Records for S1 NOT S2: 5  Relevant Records for S1 NOT S2: 0 |
| Cochrane Library | (#1 OR #2 OR #3) AND #4 AND #5; with  #1 "interprofessional cooperation":ab,ti,kw OR "interprofessional cooperative":ab,ti,kw OR "interprofessional collaboration":ab,ti,kw OR "interprofessional collaborative":ab,ti,kw OR "inter-professional cooperation":ab,ti,kw OR "inter-professional cooperative":ab,ti,kw OR "inter-professional collaboration":ab,ti,kw OR "inter-professional collaborative":ab,ti,kw OR "multi-professional collaboration":ab,ti,kw OR "multi-professional collaborative":ab,ti,kw OR "multiprofessional collaboration":ab,ti,kw OR "multiprofessional collaborative":ab,ti,kw OR "multiprofessional cooperation":ab,ti,kw OR "multiprofessional cooperative":ab,ti,kw OR "multi-professional cooperation":ab,ti,kw OR "multi-professional cooperative":ab,ti,kw OR "interdisciplinary cooperation":ab,ti,kw OR "interdisciplinary cooperative":ab,ti,kw OR "interdisciplinary collaboration":ab,ti,kw OR "interdisciplinary collaborative":ab,ti,kw OR "inter-disciplinary cooperation":ab,ti,kw OR "inter-disciplinary cooperative":ab,ti,kw OR "inter-disciplinary collaboration":ab,ti,kw OR "inter-disciplinary collaborative":ab,ti,kw OR "multi-disciplinary collaboration":ab,ti,kw OR "multi-disciplinary collaborative":ab,ti,kw OR "multidisciplinary collaboration":ab,ti,kw OR "multidisciplinary collaborative":ab,ti,kw OR "multidisciplinary cooperation":ab,ti,kw OR "multidisciplinary cooperative":ab,ti,kw OR "multi-disciplinary cooperation":ab,ti,kw OR "multi-disciplinary cooperative":ab,ti,kw OR "crossprofessional cooperation":ab,ti,kw OR "crossprofessional cooperative":ab,ti,kw OR "crossprofessional collaboration":ab,ti,kw OR "crossprofessional collaborative":ab,ti,kw OR "cross-professional cooperation":ab,ti,kw OR "cross-professional cooperative":ab,ti,kw OR "cross-professional collaboration":ab,ti,kw OR "cross-professional collaborative":ab,ti,kw OR "transprofessional cooperation":ab,ti,kw OR "transprofessional cooperative":ab,ti,kw OR "transprofessional collaboration":ab,ti,kw OR "transprofessional collaborative":ab,ti,kw OR "trans-professional cooperation":ab,ti,kw OR "trans-professional cooperative":ab,ti,kw OR "trans-professional collaboration":ab,ti,kw OR "trans-professional collaborative":ab,ti,kw OR "crossdisciplinary collaboration":ab,ti,kw OR "crossdisciplinary collaborative":ab,ti,kw OR "crossdisciplinary cooperation":ab,ti,kw OR "crossdisciplinary cooperative":ab,ti,kw OR "cross-disciplinary collaboration":ab,ti,kw OR "cross-disciplinary collaborative":ab,ti,kw OR "cross-disciplinary cooperation":ab,ti,kw OR "cross-disciplinary cooperative":ab,ti,kw OR "transdisciplinary collaboration":ab,ti,kw OR "transdisciplinary collaborative":ab,ti,kw OR "transdisciplinary cooperation":ab,ti,kw OR "transdisciplinary cooperative":ab,ti,kw OR "trans-disciplinary collaboration":ab,ti,kw OR "trans-disciplinary collaborative":ab,ti,kw OR "trans-disciplinary cooperation":ab,ti,kw OR "trans-disciplinary cooperative":ab,ti,kw  #2 = MeSH descriptor: [Interdisciplinary Communication] this term only  #3 = MeSH descriptor: [Physician-Nurse Relations] this term only  #4 = competenc*:ab,ti,kw OR capacit*:ab,ti,kw OR capabilit*:ab,ti,kw OR abilit*:ab,ti,kw OR skill*:ab,ti,kw  #5 = MeSH descriptor: [Students, Nursing] this term only | (("interprofessional cooperation" OR "interprofessional cooperative" OR "interprofessional collaboration" OR "interprofessional collaborative" OR "inter-professional cooperation" OR "inter-professional cooperative" OR "inter-professional collaboration" OR "inter-professional collaborative" OR "multi-professional collaboration" OR "multi-professional collaborative" OR "multiprofessional collaboration" OR "multiprofessional collaborative" OR "multiprofessional cooperation" OR "multiprofessional cooperative" OR "multi-professional cooperation" OR "multi-professional cooperative" OR "interdisciplinary cooperation" OR "interdisciplinary cooperative" OR "interdisciplinary collaboration" OR "interdisciplinary collaborative" OR "inter-disciplinary cooperation" OR "inter-disciplinary cooperative" OR "inter-disciplinary collaboration" OR "inter-disciplinary collaborative" OR "multi-disciplinary collaboration" OR "multi-disciplinary collaborative" OR "multidisciplinary collaboration" OR "multidisciplinary collaborative" OR "multidisciplinary cooperation" OR "multidisciplinary cooperative" OR "multi-disciplinary cooperation" OR "multi-disciplinary cooperative" OR "crossprofessional cooperation" OR "crossprofessional cooperative" OR "crossprofessional collaboration" OR "crossprofessional collaborative" OR "cross-professional cooperation" OR "cross-professional cooperative" OR "cross-professional collaboration" OR "cross-professional collaborative" OR "transprofessional cooperation" OR "transprofessional cooperative" OR "transprofessional collaboration" OR "transprofessional collaborative" OR "trans-professional cooperation" OR "trans-professional cooperative" OR "trans-professional collaboration" OR "trans-professional collaborative" OR "crossdisciplinary collaboration" OR "crossdisciplinary collaborative" OR "crossdisciplinary cooperation" OR "crossdisciplinary cooperative" OR "cross-disciplinary collaboration" OR "cross-disciplinary collaborative" OR "cross-disciplinary cooperation" OR "cross-disciplinary cooperative" OR "transdisciplinary collaboration" OR "transdisciplinary collaborative" OR "transdisciplinary cooperation" OR "transdisciplinary cooperative" OR "trans-disciplinary collaboration" OR "trans-disciplinary collaborative" OR "trans-disciplinary cooperation" OR "trans-disciplinary cooperative") AND (competenc* OR capacit* OR capabilit* OR abilit* OR skill*) AND nurs*):ab,ti,kw | Records for S1 NOT S2: 0 |
| Education Source | ((MM “Interprofessional Relations” OR AB ("interprofessional cooperation" OR "interprofessional cooperative" OR "interprofessional collaboration" OR "interprofessional collaborative" OR "inter-professional cooperation" OR "inter-professional cooperative" OR "inter-professional collaboration" OR "inter-professional collaborative" OR "multi-professional collaboration" OR "multi-professional collaborative" OR "multiprofessional collaboration" OR "multiprofessional collaborative" OR "multiprofessional cooperation" OR "multiprofessional cooperative" OR "multi-professional cooperation" OR "multi-professional cooperative" OR "interdisciplinary cooperation" OR "interdisciplinary cooperative" OR "interdisciplinary collaboration" OR "interdisciplinary collaborative" OR "inter-disciplinary cooperation" OR "inter-disciplinary cooperative" OR "inter-disciplinary collaboration" OR "inter-disciplinary collaborative" OR "multi-disciplinary collaboration" OR "multi-disciplinary collaborative" OR "multidisciplinary collaboration" OR "multidisciplinary collaborative" OR "multidisciplinary cooperation" OR "multidisciplinary cooperative" OR "multi-disciplinary cooperation" OR "multi-disciplinary cooperative" OR "crossprofessional cooperation" OR "crossprofessional cooperative" OR "crossprofessional collaboration" OR "crossprofessional collaborative" OR "cross-professional cooperation" OR "cross-professional cooperative" OR "cross-professional collaboration" OR "cross-professional collaborative" OR "transprofessional cooperation" OR "transprofessional cooperative" OR "transprofessional collaboration" OR "transprofessional collaborative" OR "trans-professional cooperation" OR "trans-professional cooperative" OR "trans-professional collaboration" OR "trans-professional collaborative" OR "crossdisciplinary collaboration" OR "crossdisciplinary collaborative" OR "crossdisciplinary cooperation" OR "crossdisciplinary cooperative" OR "cross-disciplinary collaboration" OR "cross-disciplinary collaborative" OR "cross-disciplinary cooperation" OR "cross-disciplinary cooperative" OR "transdisciplinary collaboration" OR "transdisciplinary collaborative" OR "transdisciplinary cooperation" OR "transdisciplinary cooperative" OR "trans-disciplinary collaboration" OR "trans-disciplinary collaborative" OR "trans-disciplinary cooperation" OR "trans-disciplinary cooperative") OR KW ("interprofessional cooperation" OR "interprofessional cooperative" OR "interprofessional collaboration" OR "interprofessional collaborative" OR "inter-professional cooperation" OR "inter-professional cooperative" OR "inter-professional collaboration" OR "inter-professional collaborative" OR "multi-professional collaboration" OR "multi-professional collaborative" OR "multiprofessional collaboration" OR "multiprofessional collaborative" OR "multiprofessional cooperation" OR "multiprofessional cooperative" OR "multi-professional cooperation" OR "multi-professional cooperative" OR "interdisciplinary cooperation" OR "interdisciplinary cooperative" OR "interdisciplinary collaboration" OR "interdisciplinary collaborative" OR "inter-disciplinary cooperation" OR "inter-disciplinary cooperative" OR "inter-disciplinary collaboration" OR "inter-disciplinary collaborative" OR "multi-disciplinary collaboration" OR "multi-disciplinary collaborative" OR "multidisciplinary collaboration" OR "multidisciplinary collaborative" OR "multidisciplinary cooperation" OR "multidisciplinary cooperative" OR "multi-disciplinary cooperation" OR "multi-disciplinary cooperative" OR "crossprofessional cooperation" OR "crossprofessional cooperative" OR "crossprofessional collaboration" OR "crossprofessional collaborative" OR "cross-professional cooperation" OR "cross-professional cooperative" OR "cross-professional collaboration" OR "cross-professional collaborative" OR "transprofessional cooperation" OR "transprofessional cooperative" OR "transprofessional collaboration" OR "transprofessional collaborative" OR "trans-professional cooperation" OR "trans-professional cooperative" OR "trans-professional collaboration" OR "trans-professional collaborative" OR "crossdisciplinary collaboration" OR "crossdisciplinary collaborative" OR "crossdisciplinary cooperation" OR "crossdisciplinary cooperative" OR "cross-disciplinary collaboration" OR "cross-disciplinary collaborative" OR "cross-disciplinary cooperation" OR "cross-disciplinary cooperative" OR "transdisciplinary collaboration" OR "transdisciplinary collaborative" OR "transdisciplinary cooperation" OR "transdisciplinary cooperative" OR "trans-disciplinary collaboration" OR "trans-disciplinary collaborative" OR "trans-disciplinary cooperation" OR "trans-disciplinary cooperative") OR TI ("interprofessional cooperation" OR "interprofessional cooperative" OR "interprofessional collaboration" OR "interprofessional collaborative" OR "inter-professional cooperation" OR "inter-professional cooperative" OR "inter-professional collaboration" OR "inter-professional collaborative" OR "multi-professional collaboration" OR "multi-professional collaborative" OR "multiprofessional collaboration" OR "multiprofessional collaborative" OR "multiprofessional cooperation" OR "multiprofessional cooperative" OR "multi-professional cooperation" OR "multi-professional cooperative" OR "interdisciplinary cooperation" OR "interdisciplinary cooperative" OR "interdisciplinary collaboration" OR "interdisciplinary collaborative" OR "inter-disciplinary cooperation" OR "inter-disciplinary cooperative" OR "inter-disciplinary collaboration" OR "inter-disciplinary collaborative" OR "multi-disciplinary collaboration" OR "multi-disciplinary collaborative" OR "multidisciplinary collaboration" OR "multidisciplinary collaborative" OR "multidisciplinary cooperation" OR "multidisciplinary cooperative" OR "multi-disciplinary cooperation" OR "multi-disciplinary cooperative" OR "crossprofessional cooperation" OR "crossprofessional cooperative" OR "crossprofessional collaboration" OR "crossprofessional collaborative" OR "cross-professional cooperation" OR "cross-professional cooperative" OR "cross-professional collaboration" OR "cross-professional collaborative" OR "transprofessional cooperation" OR "transprofessional cooperative" OR "transprofessional collaboration" OR "transprofessional collaborative" OR "trans-professional cooperation" OR "trans-professional cooperative" OR "trans-professional collaboration" OR "trans-professional collaborative" OR "crossdisciplinary collaboration" OR "crossdisciplinary collaborative" OR "crossdisciplinary cooperation" OR "crossdisciplinary cooperative" OR "cross-disciplinary collaboration" OR "cross-disciplinary collaborative" OR "cross-disciplinary cooperation" OR "cross-disciplinary cooperative" OR "transdisciplinary collaboration" OR "transdisciplinary collaborative" OR "transdisciplinary cooperation" OR "transdisciplinary cooperative" OR "trans-disciplinary collaboration" OR "trans-disciplinary collaborative" OR "trans-disciplinary cooperation" OR "trans-disciplinary cooperative")) AND (AB (competenc* OR capacit* OR capabilit* OR abilit* OR skill*) OR KW (competenc* OR capacit* OR capabilit* OR abilit* OR skill*) OR TI (competenc* OR capacit* OR capabilit* OR abilit* OR skill*)) AND MM “Nursing Education”) | (AB (("interprofessional cooperation" OR "interprofessional cooperative" OR "interprofessional collaboration" OR "interprofessional collaborative" OR "inter-professional cooperation" OR "inter-professional cooperative" OR "inter-professional collaboration" OR "inter-professional collaborative" OR "multi-professional collaboration" OR "multi-professional collaborative" OR "multiprofessional collaboration" OR "multiprofessional collaborative" OR "multiprofessional cooperation" OR "multiprofessional cooperative" OR "multi-professional cooperation" OR "multi-professional cooperative" OR "interdisciplinary cooperation" OR "interdisciplinary cooperative" OR "interdisciplinary collaboration" OR "interdisciplinary collaborative" OR "inter-disciplinary cooperation" OR "inter-disciplinary cooperative" OR "inter-disciplinary collaboration" OR "inter-disciplinary collaborative" OR "multi-disciplinary collaboration" OR "multi-disciplinary collaborative" OR "multidisciplinary collaboration" OR "multidisciplinary collaborative" OR "multidisciplinary cooperation" OR "multidisciplinary cooperative" OR "multi-disciplinary cooperation" OR "multi-disciplinary cooperative" OR "crossprofessional cooperation" OR "crossprofessional cooperative" OR "crossprofessional collaboration" OR "crossprofessional collaborative" OR "cross-professional cooperation" OR "cross-professional cooperative" OR "cross-professional collaboration" OR "cross-professional collaborative" OR "transprofessional cooperation" OR "transprofessional cooperative" OR "transprofessional collaboration" OR "transprofessional collaborative" OR "trans-professional cooperation" OR "trans-professional cooperative" OR "trans-professional collaboration" OR "trans-professional collaborative" OR "crossdisciplinary collaboration" OR "crossdisciplinary collaborative" OR "crossdisciplinary cooperation" OR "crossdisciplinary cooperative" OR "cross-disciplinary collaboration" OR "cross-disciplinary collaborative" OR "cross-disciplinary cooperation" OR "cross-disciplinary cooperative" OR "transdisciplinary collaboration" OR "transdisciplinary collaborative" OR "transdisciplinary cooperation" OR "transdisciplinary cooperative" OR "trans-disciplinary collaboration" OR "trans-disciplinary collaborative" OR "trans-disciplinary cooperation" OR "trans-disciplinary cooperative") AND (competenc* OR capacit* OR capabilit* OR abilit* OR skill*) AND nurs*) OR KW (("interprofessional cooperation" OR "interprofessional cooperative" OR "interprofessional collaboration" OR "interprofessional collaborative" OR "inter-professional cooperation" OR "inter-professional cooperative" OR "inter-professional collaboration" OR "inter-professional collaborative" OR "multi-professional collaboration" OR "multi-professional collaborative" OR "multiprofessional collaboration" OR "multiprofessional collaborative" OR "multiprofessional cooperation" OR "multiprofessional cooperative" OR "multi-professional cooperation" OR "multi-professional cooperative" OR "interdisciplinary cooperation" OR "interdisciplinary cooperative" OR "interdisciplinary collaboration" OR "interdisciplinary collaborative" OR "inter-disciplinary cooperation" OR "inter-disciplinary cooperative" OR "inter-disciplinary collaboration" OR "inter-disciplinary collaborative" OR "multi-disciplinary collaboration" OR "multi-disciplinary collaborative" OR "multidisciplinary collaboration" OR "multidisciplinary collaborative" OR "multidisciplinary cooperation" OR "multidisciplinary cooperative" OR "multi-disciplinary cooperation" OR "multi-disciplinary cooperative" OR "crossprofessional cooperation" OR "crossprofessional cooperative" OR "crossprofessional collaboration" OR "crossprofessional collaborative" OR "cross-professional cooperation" OR "cross-professional cooperative" OR "cross-professional collaboration" OR "cross-professional collaborative" OR "transprofessional cooperation" OR "transprofessional cooperative" OR "transprofessional collaboration" OR "transprofessional collaborative" OR "trans-professional cooperation" OR "trans-professional cooperative" OR "trans-professional collaboration" OR "trans-professional collaborative" OR "crossdisciplinary collaboration" OR "crossdisciplinary collaborative" OR "crossdisciplinary cooperation" OR "crossdisciplinary cooperative" OR "cross-disciplinary collaboration" OR "cross-disciplinary collaborative" OR "cross-disciplinary cooperation" OR "cross-disciplinary cooperative" OR "transdisciplinary collaboration" OR "transdisciplinary collaborative" OR "transdisciplinary cooperation" OR "transdisciplinary cooperative" OR "trans-disciplinary collaboration" OR "trans-disciplinary collaborative" OR "trans-disciplinary cooperation" OR "trans-disciplinary cooperative") AND (competenc* OR capacit* OR capabilit* OR abilit* OR skill*) AND nurs*) OR TI (("interprofessional cooperation" OR "interprofessional cooperative" OR "interprofessional collaboration" OR "interprofessional collaborative" OR "inter-professional cooperation" OR "inter-professional cooperative" OR "inter-professional collaboration" OR "inter-professional collaborative" OR "multi-professional collaboration" OR "multi-professional collaborative" OR "multiprofessional collaboration" OR "multiprofessional collaborative" OR "multiprofessional cooperation" OR "multiprofessional cooperative" OR "multi-professional cooperation" OR "multi-professional cooperative" OR "interdisciplinary cooperation" OR "interdisciplinary cooperative" OR "interdisciplinary collaboration" OR "interdisciplinary collaborative" OR "inter-disciplinary cooperation" OR "inter-disciplinary cooperative" OR "inter-disciplinary collaboration" OR "inter-disciplinary collaborative" OR "multi-disciplinary collaboration" OR "multi-disciplinary collaborative" OR "multidisciplinary collaboration" OR "multidisciplinary collaborative" OR "multidisciplinary cooperation" OR "multidisciplinary cooperative" OR "multi-disciplinary cooperation" OR "multi-disciplinary cooperative" OR "crossprofessional cooperation" OR "crossprofessional cooperative" OR "crossprofessional collaboration" OR "crossprofessional collaborative" OR "cross-professional cooperation" OR "cross-professional cooperative" OR "cross-professional collaboration" OR "cross-professional collaborative" OR "transprofessional cooperation" OR "transprofessional cooperative" OR "transprofessional collaboration" OR "transprofessional collaborative" OR "trans-professional cooperation" OR "trans-professional cooperative" OR "trans-professional collaboration" OR "trans-professional collaborative" OR "crossdisciplinary collaboration" OR "crossdisciplinary collaborative" OR "crossdisciplinary cooperation" OR "crossdisciplinary cooperative" OR "cross-disciplinary collaboration" OR "cross-disciplinary collaborative" OR "cross-disciplinary cooperation" OR "cross-disciplinary cooperative" OR "transdisciplinary collaboration" OR "transdisciplinary collaborative" OR "transdisciplinary cooperation" OR "transdisciplinary cooperative" OR "trans-disciplinary collaboration" OR "trans-disciplinary collaborative" OR "trans-disciplinary cooperation" OR "trans-disciplinary cooperative") AND (competenc* OR capacit* OR capabilit* OR abilit* OR skill*) AND nurs*)) | Records for S1 NOT S2: 2  Relevant Records for S1 NOT S2: 0 |
| APA PsycArticles | (MM "Competence" OR MM "Social Skills") AND (AB ("interprofessional cooperation" OR "interprofessional cooperative" OR "interprofessional collaboration" OR "interprofessional collaborative" OR "inter-professional cooperation" OR "inter-professional cooperative" OR "inter-professional collaboration" OR "inter-professional collaborative" OR "multi-professional collaboration" OR "multi-professional collaborative" OR "multiprofessional collaboration" OR "multiprofessional collaborative" OR "multiprofessional cooperation" OR "multiprofessional cooperative" OR "multi-professional cooperation" OR "multi-professional cooperative" OR "interdisciplinary cooperation" OR "interdisciplinary cooperative" OR "interdisciplinary collaboration" OR "interdisciplinary collaborative" OR "inter-disciplinary cooperation" OR "inter-disciplinary cooperative" OR "inter-disciplinary collaboration" OR "inter-disciplinary collaborative" OR "multi-disciplinary collaboration" OR "multi-disciplinary collaborative" OR "multidisciplinary collaboration" OR "multidisciplinary collaborative" OR "multidisciplinary cooperation" OR "multidisciplinary cooperative" OR "multi-disciplinary cooperation" OR "multi-disciplinary cooperative" OR "crossprofessional cooperation" OR "crossprofessional cooperative" OR "crossprofessional collaboration" OR "crossprofessional collaborative" OR "cross-professional cooperation" OR "cross-professional cooperative" OR "cross-professional collaboration" OR "cross-professional collaborative" OR "transprofessional cooperation" OR "transprofessional cooperative" OR "transprofessional collaboration" OR "transprofessional collaborative" OR "trans-professional cooperation" OR "trans-professional cooperative" OR "trans-professional collaboration" OR "trans-professional collaborative" OR "crossdisciplinary collaboration" OR "crossdisciplinary collaborative" OR "crossdisciplinary cooperation" OR "crossdisciplinary cooperative" OR "cross-disciplinary collaboration" OR "cross-disciplinary collaborative" OR "cross-disciplinary cooperation" OR "cross-disciplinary cooperative" OR "transdisciplinary collaboration" OR "transdisciplinary collaborative" OR "transdisciplinary cooperation" OR "transdisciplinary cooperative" OR "trans-disciplinary collaboration" OR "trans-disciplinary collaborative" OR "trans-disciplinary cooperation" OR "trans-disciplinary cooperative") OR KW ("interprofessional cooperation" OR "interprofessional cooperative" OR "interprofessional collaboration" OR "interprofessional collaborative" OR "inter-professional cooperation" OR "inter-professional cooperative" OR "inter-professional collaboration" OR "inter-professional collaborative" OR "multi-professional collaboration" OR "multi-professional collaborative" OR "multiprofessional collaboration" OR "multiprofessional collaborative" OR "multiprofessional cooperation" OR "multiprofessional cooperative" OR "multi-professional cooperation" OR "multi-professional cooperative" OR "interdisciplinary cooperation" OR "interdisciplinary cooperative" OR "interdisciplinary collaboration" OR "interdisciplinary collaborative" OR "inter-disciplinary cooperation" OR "inter-disciplinary cooperative" OR "inter-disciplinary collaboration" OR "inter-disciplinary collaborative" OR "multi-disciplinary collaboration" OR "multi-disciplinary collaborative" OR "multidisciplinary collaboration" OR "multidisciplinary collaborative" OR "multidisciplinary cooperation" OR "multidisciplinary cooperative" OR "multi-disciplinary cooperation" OR "multi-disciplinary cooperative" OR "crossprofessional cooperation" OR "crossprofessional cooperative" OR "crossprofessional collaboration" OR "crossprofessional collaborative" OR "cross-professional cooperation" OR "cross-professional cooperative" OR "cross-professional collaboration" OR "cross-professional collaborative" OR "transprofessional cooperation" OR "transprofessional cooperative" OR "transprofessional collaboration" OR "transprofessional collaborative" OR "trans-professional cooperation" OR "trans-professional cooperative" OR "trans-professional collaboration" OR "trans-professional collaborative" OR "crossdisciplinary collaboration" OR "crossdisciplinary collaborative" OR "crossdisciplinary cooperation" OR "crossdisciplinary cooperative" OR "cross-disciplinary collaboration" OR "cross-disciplinary collaborative" OR "cross-disciplinary cooperation" OR "cross-disciplinary cooperative" OR "transdisciplinary collaboration" OR "transdisciplinary collaborative" OR "transdisciplinary cooperation" OR "transdisciplinary cooperative" OR "trans-disciplinary collaboration" OR "trans-disciplinary collaborative" OR "trans-disciplinary cooperation" OR "trans-disciplinary cooperative") OR TI ("interprofessional cooperation" OR "interprofessional cooperative" OR "interprofessional collaboration" OR "interprofessional collaborative" OR "inter-professional cooperation" OR "inter-professional cooperative" OR "inter-professional collaboration" OR "inter-professional collaborative" OR "multi-professional collaboration" OR "multi-professional collaborative" OR "multiprofessional collaboration" OR "multiprofessional collaborative" OR "multiprofessional cooperation" OR "multiprofessional cooperative" OR "multi-professional cooperation" OR "multi-professional cooperative" OR "interdisciplinary cooperation" OR "interdisciplinary cooperative" OR "interdisciplinary collaboration" OR "interdisciplinary collaborative" OR "inter-disciplinary cooperation" OR "inter-disciplinary cooperative" OR "inter-disciplinary collaboration" OR "inter-disciplinary collaborative" OR "multi-disciplinary collaboration" OR "multi-disciplinary collaborative" OR "multidisciplinary collaboration" OR "multidisciplinary collaborative" OR "multidisciplinary cooperation" OR "multidisciplinary cooperative" OR "multi-disciplinary cooperation" OR "multi-disciplinary cooperative" OR "crossprofessional cooperation" OR "crossprofessional cooperative" OR "crossprofessional collaboration" OR "crossprofessional collaborative" OR "cross-professional cooperation" OR "cross-professional cooperative" OR "cross-professional collaboration" OR "cross-professional collaborative" OR "transprofessional cooperation" OR "transprofessional cooperative" OR "transprofessional collaboration" OR "transprofessional collaborative" OR "trans-professional cooperation" OR "trans-professional cooperative" OR "trans-professional collaboration" OR "trans-professional collaborative" OR "crossdisciplinary collaboration" OR "crossdisciplinary collaborative" OR "crossdisciplinary cooperation" OR "crossdisciplinary cooperative" OR "cross-disciplinary collaboration" OR "cross-disciplinary collaborative" OR "cross-disciplinary cooperation" OR "cross-disciplinary cooperative" OR "transdisciplinary collaboration" OR "transdisciplinary collaborative" OR "transdisciplinary cooperation" OR "transdisciplinary cooperative" OR "trans-disciplinary collaboration" OR "trans-disciplinary collaborative" OR "trans-disciplinary cooperation" OR "trans-disciplinary cooperative")) AND MM "Nursing Education" | AB (("interprofessional cooperation" OR "interprofessional cooperative" OR "interprofessional collaboration" OR "interprofessional collaborative" OR "inter-professional cooperation" OR "inter-professional cooperative" OR "inter-professional collaboration" OR "inter-professional collaborative" OR "multi-professional collaboration" OR "multi-professional collaborative" OR "multiprofessional collaboration" OR "multiprofessional collaborative" OR "multiprofessional cooperation" OR "multiprofessional cooperative" OR "multi-professional cooperation" OR "multi-professional cooperative" OR "interdisciplinary cooperation" OR "interdisciplinary cooperative" OR "interdisciplinary collaboration" OR "interdisciplinary collaborative" OR "inter-disciplinary cooperation" OR "inter-disciplinary cooperative" OR "inter-disciplinary collaboration" OR "inter-disciplinary collaborative" OR "multi-disciplinary collaboration" OR "multi-disciplinary collaborative" OR "multidisciplinary collaboration" OR "multidisciplinary collaborative" OR "multidisciplinary cooperation" OR "multidisciplinary cooperative" OR "multi-disciplinary cooperation" OR "multi-disciplinary cooperative" OR "crossprofessional cooperation" OR "crossprofessional cooperative" OR "crossprofessional collaboration" OR "crossprofessional collaborative" OR "cross-professional cooperation" OR "cross-professional cooperative" OR "cross-professional collaboration" OR "cross-professional collaborative" OR "transprofessional cooperation" OR "transprofessional cooperative" OR "transprofessional collaboration" OR "transprofessional collaborative" OR "trans-professional cooperation" OR "trans-professional cooperative" OR "trans-professional collaboration" OR "trans-professional collaborative" OR "crossdisciplinary collaboration" OR "crossdisciplinary collaborative" OR "crossdisciplinary cooperation" OR "crossdisciplinary cooperative" OR "cross-disciplinary collaboration" OR "cross-disciplinary collaborative" OR "cross-disciplinary cooperation" OR "cross-disciplinary cooperative" OR "transdisciplinary collaboration" OR "transdisciplinary collaborative" OR "transdisciplinary cooperation" OR "transdisciplinary cooperative" OR "trans-disciplinary collaboration" OR "trans-disciplinary collaborative" OR "trans-disciplinary cooperation" OR "trans-disciplinary cooperative") AND (competenc* OR capacit* OR capabilit* OR abilit* OR skill*) AND nurs*) OR TI (("interprofessional cooperation" OR "interprofessional cooperative" OR "interprofessional collaboration" OR "interprofessional collaborative" OR "inter-professional cooperation" OR "inter-professional cooperative" OR "inter-professional collaboration" OR "inter-professional collaborative" OR "multi-professional collaboration" OR "multi-professional collaborative" OR "multiprofessional collaboration" OR "multiprofessional collaborative" OR "multiprofessional cooperation" OR "multiprofessional cooperative" OR "multi-professional cooperation" OR "multi-professional cooperative" OR "interdisciplinary cooperation" OR "interdisciplinary cooperative" OR "interdisciplinary collaboration" OR "interdisciplinary collaborative" OR "inter-disciplinary cooperation" OR "inter-disciplinary cooperative" OR "inter-disciplinary collaboration" OR "inter-disciplinary collaborative" OR "multi-disciplinary collaboration" OR "multi-disciplinary collaborative" OR "multidisciplinary collaboration" OR "multidisciplinary collaborative" OR "multidisciplinary cooperation" OR "multidisciplinary cooperative" OR "multi-disciplinary cooperation" OR "multi-disciplinary cooperative" OR "crossprofessional cooperation" OR "crossprofessional cooperative" OR "crossprofessional collaboration" OR "crossprofessional collaborative" OR "cross-professional cooperation" OR "cross-professional cooperative" OR "cross-professional collaboration" OR "cross-professional collaborative" OR "transprofessional cooperation" OR "transprofessional cooperative" OR "transprofessional collaboration" OR "transprofessional collaborative" OR "trans-professional cooperation" OR "trans-professional cooperative" OR "trans-professional collaboration" OR "trans-professional collaborative" OR "crossdisciplinary collaboration" OR "crossdisciplinary collaborative" OR "crossdisciplinary cooperation" OR "crossdisciplinary cooperative" OR "cross-disciplinary collaboration" OR "cross-disciplinary collaborative" OR "cross-disciplinary cooperation" OR "cross-disciplinary cooperative" OR "transdisciplinary collaboration" OR "transdisciplinary collaborative" OR "transdisciplinary cooperation" OR "transdisciplinary cooperative" OR "trans-disciplinary collaboration" OR "trans-disciplinary collaborative" OR "trans-disciplinary cooperation" OR "trans-disciplinary cooperative") AND (competenc* OR capacit* OR capabilit* OR abilit* OR skill*) AND nurs*) OR KW (("interprofessional cooperation" OR "interprofessional cooperative" OR "interprofessional collaboration" OR "interprofessional collaborative" OR "inter-professional cooperation" OR "inter-professional cooperative" OR "inter-professional collaboration" OR "inter-professional collaborative" OR "multi-professional collaboration" OR "multi-professional collaborative" OR "multiprofessional collaboration" OR "multiprofessional collaborative" OR "multiprofessional cooperation" OR "multiprofessional cooperative" OR "multi-professional cooperation" OR "multi-professional cooperative" OR "interdisciplinary cooperation" OR "interdisciplinary cooperative" OR "interdisciplinary collaboration" OR "interdisciplinary collaborative" OR "inter-disciplinary cooperation" OR "inter-disciplinary cooperative" OR "inter-disciplinary collaboration" OR "inter-disciplinary collaborative" OR "multi-disciplinary collaboration" OR "multi-disciplinary collaborative" OR "multidisciplinary collaboration" OR "multidisciplinary collaborative" OR "multidisciplinary cooperation" OR "multidisciplinary cooperative" OR "multi-disciplinary cooperation" OR "multi-disciplinary cooperative" OR "crossprofessional cooperation" OR "crossprofessional cooperative" OR "crossprofessional collaboration" OR "crossprofessional collaborative" OR "cross-professional cooperation" OR "cross-professional cooperative" OR "cross-professional collaboration" OR "cross-professional collaborative" OR "transprofessional cooperation" OR "transprofessional cooperative" OR "transprofessional collaboration" OR "transprofessional collaborative" OR "trans-professional cooperation" OR "trans-professional cooperative" OR "trans-professional collaboration" OR "trans-professional collaborative" OR "crossdisciplinary collaboration" OR "crossdisciplinary collaborative" OR "crossdisciplinary cooperation" OR "crossdisciplinary cooperative" OR "cross-disciplinary collaboration" OR "cross-disciplinary collaborative" OR "cross-disciplinary cooperation" OR "cross-disciplinary cooperative" OR "transdisciplinary collaboration" OR "transdisciplinary collaborative" OR "transdisciplinary cooperation" OR "transdisciplinary cooperative" OR "trans-disciplinary collaboration" OR "trans-disciplinary collaborative" OR "trans-disciplinary cooperation" OR "trans-disciplinary cooperative") AND (competenc* OR capacit* OR capabilit* OR abilit* OR skill*) AND nurs*) | Records for S1 NOT S2: 0 |

Note: Due to the heterogeneous indexing systems and thesaurus systems across the individual ProQuest databases, no controlled vocabulary adaptation was applied. The other databases included in the final search strategy but not listed in this table do not provide controlled vocabulary.
