# Supplementary material for: Shortcomings of modeling interprofessional collaboration competence in nurse education: A protocol for a scoping review of empirical research
Source: MethodsX. 2026 Aug 4;17:104089. doi: 10.1016/j.mex.2026.104089 (PMC13474387; doi:10.1016/j.mex.2026.104089)
Supplement: Supplementary file 3 [file mmc3.docx]

**Python scripts supporting intercoder agreement – Python 3.14.5**

**Title and abstract screening**

```
import pandas as pd
from sklearn.metrics import cohen_kappa_score

# ----------------------------------------
# Load CSV files exported from Zotero
# ----------------------------------------

reviewer_a = pd.read_csv(r[Enter the full path for the document “Reviewer 1_title and abstract screening”])
reviewer_b = pd.read_csv(r[Enter the full path for the document “Reviewer 2_title and abstract screening”])

# ----------------------------------------
# Extract ASReview screening labels
# ----------------------------------------

reviewer_a["label"] = reviewer_a["Notes"].str.extract(
    r"(ASReview_relevant|ASReview_irrelevant)"
)

reviewer_b["label"] = reviewer_b["Notes"].str.extract(
    r"(ASReview_relevant|ASReview_irrelevant)"
)

# ----------------------------------------
# Convert labels to binary values
# ----------------------------------------

label_mapping = {
    "ASReview_relevant": 1,
    "ASReview_irrelevant": 0
}

reviewer_a["label"] = reviewer_a["label"].map(label_mapping)
reviewer_b["label"] = reviewer_b["label"].map(label_mapping)

# ----------------------------------------
# Merge datasets based on study title
# because DOI is not available in any case
# ----------------------------------------

merged = reviewer_a[["Title", "label"]].merge(
    reviewer_b[["Title", "label"]],
    on="Title",
    suffixes=("_A", "_B")
)

# ----------------------------------------
# Plausibility check: how many studies are being compared
# ----------------------------------------

print(f"Number of studies compared: {len(merged)}")

# ----------------------------------------
# Create agreement matrix
# ----------------------------------------

agreement_matrix = pd.crosstab(
    merged["label_A"],
    merged["label_B"],
    rownames=["Reviewer A"],
    colnames=["Reviewer B"]
)

print("\nAgreement matrix:")
print(agreement_matrix)
```

```
# ----------------------------------------
# Studies classified as relevant by both reviewers
# ----------------------------------------

both_relevant = merged[
    (merged["label_A"] == 1) &
    (merged["label_B"] == 1)
]

# ----------------------------------------
# Studies classified as irrelevant by both reviewers
# ----------------------------------------

both_irrelevant = merged[
    (merged["label_A"] == 0) &
    (merged["label_B"] == 0)
]

# ----------------------------------------
# Studies classified as relevant by Reviewer A and irrelevant by Reviewer B
# ----------------------------------------

a_only = merged[
    (merged["label_A"] == 1) &
    (merged["label_B"] == 0)
]

# ----------------------------------------
# Studies classified as irrelevant by Reviewer A and relevant by Reviewer B
# ----------------------------------------

b_only = merged[
    (merged["label_A"] == 0) &
    (merged["label_B"] == 1)
]

# ----------------------------------------
# Calculate Cohen's Kappa
# ----------------------------------------

kappa = cohen_kappa_score(
    merged["label_A"],
    merged["label_B"]
)

# ----------------------------------------
# Output: Cohen’s kappa
# ----------------------------------------

print(f"Cohen's kappa: {kappa:.3f}")

# ----------------------------------------
# Export study titles for each cell of the agreement matrix to separate Excel sheets
# ----------------------------------------

with pd.ExcelWriter("screening_disagreements.xlsx") as writer:
    both_relevant.to_excel(writer, sheet_name="Both_Relevant", index=False)
    both_irrelevant.to_excel(writer, sheet_name="Both_Irrelevant", index=False)
    a_only.to_excel(writer, sheet_name="A_Only", index=False)
    b_only.to_excel(writer, sheet_name="B_Only", index=False)
```

**Full-Text screening**

```
import pandas as pd
from sklearn.metrics import cohen_kappa_score

# ----------------------------------------
# Load CSV files exported from Zotero
# ----------------------------------------

reviewer_a = pd.read_csv(r"[Enter the full path for the document “Reviewer 1_full-text screening.csv”]")
reviewer_b = pd.read_csv(r"[Enter the full path for the document “Reviewer 2_full-text screening.csv”]")

# ----------------------------------------
# Records marked with "Full-text screening: Not relevant" are coded as not relevant.
# Records without this note are coded as relevant.
# ----------------------------------------

exclusion_note = "full-text screening: not relevant"
reviewer_a["label"] = reviewer_a["Notes"].fillna("").str.lower().apply(
    lambda x: 0 if exclusion_note in x else 1
)
reviewer_b["label"] = reviewer_b["Notes"].fillna("").str.lower().apply(
    lambda x: 0 if exclusion_note in x else 1
)

# ----------------------------------------
# Merge datasets based on study title
# because DOI is not available in any case
# ----------------------------------------

merged = reviewer_a[["Title", "label"]].merge(
    reviewer_b[["Title", "label"]],
    on="Title",
    suffixes=("_A", "_B")
)

# ----------------------------------------
# Plausibility check: how many studies are being compared
# ----------------------------------------

print(f"Number of full-text records compared: {len(merged)}")

# ----------------------------------------
# Create agreement matrix
# ----------------------------------------

agreement_matrix = pd.crosstab(
    merged["label_A"],
    merged["label_B"],
    rownames=["Reviewer A"],
    colnames=["Reviewer B"]
)
print("\nAgreement matrix:")
print(agreement_matrix)

# ----------------------------------------
# Full-text records classified as relevant by both reviewers
# ----------------------------------------

both_relevant = merged[
    (merged["label_A"] == 1) &
    (merged["label_B"] == 1)
]
```

```
# ----------------------------------------
# Full-text records classified as not relevant by both reviewers
# ----------------------------------------

both_not_relevant = merged[
    (merged["label_A"] == 0) &
    (merged["label_B"] == 0)
]

# ----------------------------------------
# Full-text records classified as relevant by Reviewer A and not relevant by Reviewer B
# ----------------------------------------

a_only = merged[
    (merged["label_A"] == 1) &
    (merged["label_B"] == 0)
]

# ----------------------------------------
# Full-text records classified as not relevant by Reviewer A and relevant by Reviewer B
# ----------------------------------------

b_only = merged[
    (merged["label_A"] == 0) &
    (merged["label_B"] == 1)
]

# ----------------------------------------
# Calculate Cohen's Kappa
# ----------------------------------------

kappa = cohen_kappa_score(
    merged["label_A"],
    merged["label_B"]
)

# ----------------------------------------
# Output: Cohen’s kappa
# ----------------------------------------

print(f"Cohen's kappa for full-text screening: {kappa:.3f}")

# ----------------------------------------
# Export study titles for each cell of the agreement matrix to separate Excel sheets
# ----------------------------------------

with pd.ExcelWriter("full_text_screening_agreement_matrix.xlsx") as writer:
    both_relevant.to_excel(writer, sheet_name="Both_Relevant", index=False)
    both_not_relevant.to_excel(writer, sheet_name="Both_Not_Relevant", index=False)
    a_only.to_excel(writer, sheet_name="A_Relevant_B_Not", index=False)
    b_only.to_excel(writer, sheet_name="A_Not_B_Relevant", index=False)
```
