# Supplementary material for: Shortcomings of modeling interprofessional collaboration competence in nurse education: A protocol for a scoping review of empirical research
Source: MethodsX. 2026 Aug 4;17:104089. doi: 10.1016/j.mex.2026.104089 (PMC13474387; doi:10.1016/j.mex.2026.104089)
Supplement: Supplementary file 4 [file mmc4.docx]

**Data extraction form with completed examples from pilot studies**

| **Ref No.** | **Author(s) (Year)** | **Country &**  **Setting** | **Study design** | **Study aim(s)** | **Group and size** | **Focused facet(s) of competence & Instrument (if applicable)** | **Main qualitative findings** | **Extent of theory talk** |
| --- | --- | --- | --- | --- | --- | --- | --- | --- |
| Quantitative | | | | | | | | |
| 1 | Baker and Durham (2013) | *Country*: USA  *Setting*: Academic; undergraduate | Quasi-experimental | To examine students’ ICC after participation in a TeamSTEPPS-based interprofessional education course | Total: 17 students (nursing, pharmacy and medicine) | Affect-motivation*  *Instrument*: Interprofessional Collaborative Competencies Attainment Scale (ICCAS) | Not applicable | None |
| 2 | Hartnett et al. (2019) | *Country*: USA  *Setting*: Academic; Not reported | Quasi-experimental | To evaluate the effectiveness of an innovative pediatric interprofessional education clinical experience using oral-systemic health to improve self-reported interprofessional competencies | Total: 159 (55 family nurse practitioner, 59 dental and 45 medical students) | Affect-motivation*  *Instrument*: Interprofessional Collaborative Competencies Attainment Scale (ICCAS) | Not applicable | None |
| Mixed methods | | | | | | | | |
| 3 | Champagne-Langabeer et al. (2019) | *Country*: USA  Setting: Academic; undergraduate & postgraduate | Quantitatively driven | To evaluate the change in pre- versus post-collaboration scores for students who participated in an IPE simulation | Total: 408  Medical School: 198  Nursing: 157  Biomedical Informatics: 16  Dental: 22  Public Health: 15 | Affect-motivation*  *Instrument*: Interprofessional Collaborative Competencies Attainment Scale (ICCAS) | Students reported that both simulations allowed them to better understand their own role and the roles of the collaboration partners | None |
| 4 | Gibbons et al. (2022) | *Country*: Canada  Setting: Academic; undergraduate & postgraduate | Qualitatively driven | To identify the interprofessional collaboration competencies developed during a home palliative care simulation with standardized patients and family members | Total: 14 (6 nursing students, 5 nurse practitioner students, and 3 paramedics) | Affect-motivation*  *Instrument*: Interprofessional Collaborator Assessment Rubric (self-reported)  Performance  Instrument: Interprofessional Collaborator Assessment Rubric (ratings by researchers) | The self-assessment of ten participants were higher than the scores assigned by the researchers | Low |

Note: * = Based on the instruments used to measure nursing students’ ICC (see Blömeke 2017), as no further descriptions of the focused competence facet(s) were provided.

**References**

Baker, M. J., & Durham, C. F. (2013). Interprofessional education: A survey of students’ collaborative competency outcomes. *Journal of Nursing Education, 52*(12), 713–718. <https://doi.org/10.3928/01484834-20131118-04>

Blömeke, S. (2017). Assuring quality in competence assessments: The value added of applying different assessment approaches to professional education. In M. Mulder (Ed.), *Competency-based vocational and professional education* (pp. 631–648). Springer. <https://doi.org/10.1007/978-3-319-41713-4_29>

Champagne-Langabeer, T., Revere, L., Tankimovich, M., Yu, E., Spears, R., & Swails, J. L. (2019). Integrating diverse disciplines to enhance interprofessional competency in healthcare delivery. *Healthcare, 7*(2), 75. <https://doi.org/10.3390/healthcare7020075>

Gibbons, C., Landry, V., & Boudreau, S. (2022). Interprofessional collaboration competencies of nursing students, nurse practitioner students, and paramedics in a simulated palliative home care setting: A pilot study. *Quality Advancement in Nursing Education – Avancées en formation infirmière, 8*(4), 3. <https://doi.org/10.17483/2368-6669.1340>

Hartnett, E., Haber, J., Catapano, P., Dougherty, N., Moursi, A. M., Kashani, R., Osman, C., Chinn, C., & Bella, A. (2019). The impact of an interprofessional pediatric oral health clerkship on advancing interprofessional education outcomes. *Journal of Dental Education, 83*(8), 878–886. <https://doi.org/10.21815/JDE.019.088>
