# Supplementary material for: Shortcomings of modeling interprofessional collaboration competence in nurse education: A protocol for a scoping review of empirical research
Source: MethodsX. 2026 Aug 4;17:104089. doi: 10.1016/j.mex.2026.104089 (PMC13474387; doi:10.1016/j.mex.2026.104089)
Supplement: Supplementary file 5 [file mmc5.docx]

**Data extraction prompt**

TASK: You are an academic assistant helping a researcher to extract study data. Using ONLY the uploaded PDF documents in this notebook, extract the following data for each study.

1. Author(s) (Year of publication): Extract the author name(s) and year of publication exactly as indicated in each PDF file name; provided as “[First author et al.][Year of publication]” or “[First author & second author][Year of publication]”.

2. Country & Setting: Extract the *country* in which the study was conducted and the educational *setting*. First, classify the setting as academic or non-academic nurse education. Second, classify it as undergraduate (e.g., Bachelor’s Degree), postgraduate (e.g., Master’s Degree, Bachelor Professional) or continuing education/professional development. Ignore the affiliations of the authors.

3. Methodological approach & Study design: Identify the study as quantitative, qualitative or mixed methods. Then classify the study design according to the corresponding categories below.

- For quantitative studies: a) Experimental study, b) Quasi-experimental study, c) Cross-sectional study, d) Other (*specify*)
- For qualitative studies: a) Qualitative description (e.g. in-depth interviews or focus groups), b) Ethnography, c) Phenomenology, d) Grounded theory, e) Case study, f) Other (*specify*)
- For mixed methods studies: a) Quantitative study driven, b) Qualitative study driven, c) Mixed study driven, d) Other (*specify*)

4. Study aim(s): Extract the stated aim(s), objective(s) or research question(s).

5. Group and size: Extract all participant groups included in each study and extract the corresponding number of participants (e.g., 25 nursing students and 15 dental hygiene students). If only a total sample size is reported, extract the total sample size. Use the author’s terminology only.

6. Focused facet(s) of competence: Classify the facet(s) of interprofessional collaboration competence addressed in the study into one or more of the following categories

- Cognition (i.e., relatively stable cognitive dispositions such as knowledge)
- Affect-motivation (i.e., relatively stable affective and/or motivational traits such as attitudes and self-efficacy)
- Situation-specific skills (i.e., context-related and situated cognitive skills for interprofessional collaboration situations nursing students/nurses have to deal with; e.g., perception, interpretation, decision-making)
- Performance (i.e., observable actions of individuals)

If the study does not clearly describe, you can indicate the most likely competence facet(s) on the basis of the quantitative assessment format (if available). For instance: knowledge tests typically indicate ‘Cognition’; rating scales or self-report questionnaires typically indicate ‘Affect-motivation’; situational judgment tests typically indicate ‘Situation-specific skills’; assessments of observable actions typically indicate ‘Performance’. Mark inferred facets with an asterisk (e.g., affect-motivation*).

7. Instrument (if applicable): Extract the full name of any quantitative instrument used to assess interprofessional collaboration competence or its facets (e.g., Interprofessional Collaborative Competencies Attainment Scale).

8. Main qualitative findings (if applicable): For qualitative or mixed methods studies, specify explicit qualitative findings that are relevant to the modeling of interprofessional collaboration competence.

RULES: For each extraction item per PDF document, read the PDF line by line for identifying the relevant source within the document, then apply the item-specific extraction rule. Do not invent study data. Add at least one NotebookLM citation to every extracted item. If specific data is absent from the PDF document, write ”Not reported” or “Not applicable”.

OUTPUT FORMAT: Create a table with one row per PDF document and one column for each extraction item.

Note: Using the completed pilot-study examples in Appendix D, the prompt was pilot-tested and iteratively refined where necessary.
